# Microencapsulation of Chokeberry Polyphenols and Volatiles: Application of Alginate and Pectin as Wall Materials

**DOI:** 10.3390/gels7040231

**Published:** 2021-11-24

**Authors:** Ina Ćorković, Anita Pichler, Ivana Ivić, Josip Šimunović, Mirela Kopjar

**Affiliations:** 1 Josip Juraj Strossmayer University, Faculty of Food Technology, F. Kuhača 18, 31000 Osijek, Croatia; ina.corkovic@ptfos.hr (I.Ć.); anita.pichler@ptfos.hr (A.P.); ivana.ivic@ptfos.hr (I.I.); 2 North Carolina State University, Department of Food, Bioprocessing and Nutrition Sciences, Raleigh, NC 27695-7624, USA; simun@ncsu.edu

**Keywords:** microencapsulation, alginate, pectin, beads, polyphenols, volatiles

## Abstract

Microencapsulation is a rapidly evolving technology that allows preservation of various high-value, but unstable, compounds, such as polyphenols and volatiles. These components of chokeberry juice are reported to have various health-promoting properties. In the present study, hydrogel beads with alginate or alginate and pectin as wall materials and chokeberry juice as active agent were prepared using Encapsulator B-390. The effects of different compositions of wall material as well as the duration of complexation (30 or 90 min) with hardening solution on microencapsulation of chokeberry polyphenols and volatiles were investigated. Spectrophotometric and HPLC analyses showed that beads with pectin addition contained higher concentrations of polyphenols and anthocyanins compared to those prepared with alginate. Antioxidant activities evaluated with FRAP, CUPRAC, DPPH, and ABTS assays followed the same trend. Encapsulation of volatiles which were determined using GC-MS analysis also depended on the composition of hydrogel beads and in some cases on the time of complexation. Results of this study showed that the selection of the wall material is a relevant factor determining the preservation of polyphenols and volatiles. The incorporation of bioactive compounds in hydrogel beads opens up a wide range of possibilities for the development of functional and innovative foods.

## 1. Introduction

Microencapsulation is a process that has developed rapidly and is widely used in the food, cosmetics, and medical fields [1]. It enhances the stability of an active agent, allows its controlled release, and flavor masking. As polyphenols and volatiles have similar unstable properties, various encapsulation techniques are applied nowadays to achieve their preservation [2,3]. Encapsulation is a process in which substance or an active agent is entrapped into wall material [4]. To date, different encapsulation techniques have been developed, such as physical (spray-drying, freeze-drying and electrospraying), chemical (molecular inclusion) and physicochemical methods (ionic gelation and coacervation) to improve the physical properties of bioactive compounds, prevent their degradation reactions, prolong their shelf-life, or provide a targeted release [5]. One of the commercially available encapsulators for the production of hydrogel beads is BÜCHI Encapsulator B-390. The mixture consisting of the encapsulating substance dissolved in a polymer solution is pumped by air pressure and, after the formation of a laminar liquid stream, this device uses superimposed vibration to break it [6]. Beads are gelified upon landing in a bath of calcium chloride. After formation, they can be further processed by adding a new membrane, and then directly used or stored. Beads formed using this technique can protect the encapsulant from external factors, improve its organoleptic properties, or enable its controlled release [7]. This area is an active field of research since encapsulation of polyphenols and volatiles with polysaccharides as wall materials have been previously investigated [8,9,10,11,12,13]. Pectin, alginate, starch, and carrageenan are commonly used materials in microencapsulation processes as the U.S. Food and Drug Administration regards them as GRAS [10]. Pectin is one of the most important polysaccharides used in the food industry due to its gelling, thickening, and emulsification characteristics. Its chemical composition affects its properties, but the content of methylated carboxyl functional groups determines the gelling ability of pectin [14]. Pectin with a low esterification degree is able to form a polymer network in the presence of Ca^2+^ ions. Presence of cations determines the gelling ability of alginate, similar to pectin. Alginate chains in the presence of divalent cations interact with the ions and cross-linking with the nearby chains occurs. Formation of hydrogel structures enables the controlled release of bioactives in the food and pharmaceutical industries [15]. In the present study, chokeberry juice was selected as a source of polyphenols and volatiles. Due to the high concentrations of bioactive compounds, consumption of chokeberry juice is associated with various positive effects on human health, such as antioxidant, anti-inflammatory, antimutagenic, gastroprotective and hepatoprotective activities [16].

We applied the microencapsulation technique using vibration technology to produce alginate (ALG) and alginate/pectin (ALG/PEC) beads from chokeberry juice. Effects of different wall materials and duration of complexation (30 or 90 min) with a hardening solution on total polyphenols and anthocyanins (both spectrophotometrically and using HPLC method), antioxidant activity (FRAP, CUPRAC, DPPH, and ABTS assays) and volatile profiles of hydrogel beads prepared from chokeberry juice were investigated.

## 2. Results and Discussion

### 2.1. Polyphenols and Antioxidant Activity of Chokeberry Juice

Chokeberry juice used for the preparation of hydrogel beads was evaluated. Total polyphenols, monomeric anthocyanins, antioxidant activity (FRAP, CUPRAC, DPPH, ABTS assay) and concentrations of individual polyphenols were determined using the HPLC method and are presented in Table 1.

For the purpose of this study, four different samples were prepared. ALG and ALG/PEC abbreviations denote whether only alginate or alginate and pectin combined were used as the wall material(s). The number next to the abbreviation denotes the time of complexation with hardening solution (30 or 90 min).

### 2.2. Evaluation of Polyphenols

Results of total polyphenols and anthocyanins encapsulated in hydrogel beads are presented in Table 2. Total polyphenols of hydrogel beads ranged from 4.44 mg/g to 5.66 mg/g. It was observed that the time of complexation did not affect encapsulation of total polyphenols, while the addition of pectin to wall material caused its increase. As opposed to total polyphenols, prolonged complexation caused changes in the concentration of monomeric anthocyanins. In the case of ALG beads, longer complexation caused a decrease in anthocyanin concentration, while in the case of ALG/PEC beads it had a positive effect. The lowest ability to encapsulate anthocyanins was by the ALG-90 sample (1.29 g/kg), while the highest was observed for the ALG/PEC-90 (1.47 g/kg). Concentrations of individual polyphenols in hydrogel beads were evaluated by the HPLC method and these concentrations are presented in Table 3. In the encapsulation process, entrapment of individual polyphenols depended on the composition of the wall material [17]. Detected polyphenols in chokeberry juice were cyanidin-3-galactoside, cyanidin-3-arabinoside, cyanidin-3-xyloside, chlorogenic acid, neochlorogenic acid, quercetin, hyperoside and rutin (Table 1). These findings were in accordance with the previously published literature [18,19,20]. Polyphenols present in chokeberry juice were also present in the prepared hydrogel beads, except for the absence of cyanidin-3-xyloside. The most abundant anthocyanin in chokeberry juice was cyanidin-3-galactoside as well as in the hydrogel beads, while cyanidin-3-arabinoside was present in hydrogel beads in lower concentrations. Both of these anthocyanins were detected in higher concentrations in ALG/PEC hydrogel beads. Detected phenolic acids were chlorogenic and neochlorogenic acid. Addition of pectin was proven to be more effective in encapsulation of chlorogenic acid while this was not the case for neochlorogenic acid. Concentration of chlorogenic acid was 306.25 mg/kg for ALG samples and 319.94 mg/kg for ALG/PEC samples, while ALG samples had higher concentrations of neochlorogenic acid (174.69 mg/kg for ALG samples and 142.28 mg/kg for ALG/PEC samples). Time of complexation did not affect their encapsulation. Detected flavonols in beads were quercetin, hyperoside, and rutin. Among them, rutin was the most abundant. Rutin concentration was about 35.48 mg/kg in all samples. The addition of pectin to wall material caused an increase in quercetin concentration in hydrogel beads, while prolonged complexation did not affect its encapsulation. The concentration of hyperoside in hydrogel beads was about 24.75 mg/kg.

It was observed that the addition of pectin to the wall material of hydrogel beads was a useful approach for the encapsulation of polyphenols since ALG/PEC samples had a higher concentration of total polyphenols than ALG samples. In the study of Bušić et al. [9] it was reported that the addition of other polysaccharides (pectin, cellulose derivates and chitosan) or fillers (carob powder or cocoa) to alginate enhanced the encapsulation of polyphenols by the improvement of the alginate porous structure which enabled the encapsulation of compounds with a lower molecular weight. The combination of alginate and pectin with proteins or cellulose derivates ensured the encapsulation of polyphenols by reducing the effective diffusivity through alginate hydrogel microstructure where additional components served as a solid barrier that blocked the transport from the beads to the surroundings.

Possible interactions between components that ensured the chemical entrapment of bioactives in the bead’s matrix is another explanation. Additionally, reinforcement of pectin and alginate beads could be achieved by using a combination of other polysaccharides or proteins with alginate and pectin because ionically cross-linked pectin and alginate hydrogels are not homogeneous as a result of the cluster formation of ionically associated domains [17]. Aguirre Calvo et al. [11] studied the encapsulation of polyphenols in hydrogel beads prepared with alginate alone or in combination with different gums and pectin. Total polyphenols of beads prepared from beetroot extracts followed the order: guar gum < low methoxyl pectin, high methoxyl pectin, alginate < Arabic gum. The reductions of total polyphenols in alginate/pectin beads were explained by the presence of additional polygalacturonate groups that interacted with polyphenols and promoted modifications of associations between alginate and Ca^2+^. However, if alginate was used as the only constituent of the bead wall material, its higher concentration caused better preservation and encapsulation of polyphenols [8,13].

In our study, extension of complexation from 30 to 90 min did not affect the encapsulation of total polyphenols. The effect of the complexation time of polysaccharides with calcium ions has been investigated in other studies as well. Zam et al. [21] claimed that upon calcium and alginate contact, formation of the gel occurred directly at the interface, but the homogeneity of the matrix depended on the calcium diffusion. It was reported that if the exposure of the alginate to the calcium ions is prolonged, more calcium will diffuse into the gel network and bind to the alginate. However, longer times could cause the release of polyphenols to the matrix or the shift of the bounded calcium ions by alginate. In hydrogel beads, prolonged complexation had a positive effect on the concentration of chlorogenic acid and the same effect was observed after the addition of pectin to the wall material. Its concentration was higher in hydrogel beads compared to the concentration of neochlorogenic acid. The same effect was observed in the study of Padayachee et al. [22], where chlorogenic acid from purple carrot juice was always found as the major phenolic acid in solution after contact with pectin and cellulose composites. However, similar conditions caused quite the opposite effect on the concentration of neochlorogenic acid. It can be concluded that molecular weight is not the only factor affecting the binding of phenolic acids to polysaccharides, as was reported previously since these two compounds have identical molecular weights [22]. The number and the position of hydroxyl groups of phenolic acids were other factors affecting the interactions via covalent and hydrogen bonds with polysaccharides [17]. Since food is a complex matrix, phenolic acids can interact with each other or with other food components and thus their properties can be changed. High encapsulation of rutin in pectin beads was also observed in the study of Jantrawut et al. [1]. This was explained by the low solubility of rutin in cross-linking solution. In addition, texture properties, such as the rigidity of gel, were associated with its encapsulation. The presence of sugars and hydroxyl groups at different positions in the B ring of flavonols caused changes in their interactions with polysaccharides [23]. Another factor determining the encapsulation of compounds inside alginate beads is their polarity. For more hydrophobic polyphenols, poor encapsulation occurs due to non-incorporation into the encapsulation mixture, which usually consists of hydrophilic materials, such as alginate or pectin, and thus their encapsulation remains a challenge [24].

In the study of Guo et al. [10], the major loss of anthocyanins was described as a consequence of curing before gelling from liquid droplet to hardening solution. It was suggested that higher concentrations of total polysaccharides could cause better preservation of anthocyanins, as was observed in the present study when pectin was added. In general, diffusion of anthocyanins within the hydrogel particles is considered as diffusion in porous media and it is associated with the porosity of the hydrogel beads. Hence the additional polysaccharides increase the gelation rate before the hydrogels are set and reduce the diffusion of anthocyanins into the environment by decreasing the porosity after hydrogel formation. Microencapsulation of anthocyanins was affected by physicochemical interactions and the formation of covalent bonds between alginate-pectin and anthocyanins [10]. In addition to the physical boundary formation, interactions between pectin-alginate and anthocyanins may lead to increased stability of anthocyanins. Both alginate and pectin are polyuronic acids and electrostatic interactions between the anthocyanin flavylium cation and the dissociated carboxylic groups of the polysaccharides caused a stabilization effect similar to the binding of calcium ions in pectin and alginate gels [25]. For a better understanding of anthocyanin–polysaccharide interactions, it is important to note that, initially, their direct mutual interaction occurs and the subsequent binding includes the formation of a stacking effect, i.e., interactions of additional anthocyanins with those already bonded [26].

Encapsulation efficiency was also determined by dividing the concentration of polyphenols in hydrogel beads with the initial concentration of polyphenols and multiplying by 100. Encapsulation efficiency of total polyphenols in ALG hydrogel beads was around 39%, while in the ALG/PEC beads was higher, around 50%. Values were higher for anthocyanins encapsulation for which we observed that ALG-30 hydrogel beads had 72% and ALG-90 69% of encapsulation efficiency, while for ALG/PEC encapsulation efficiency was 76%. Lower values of encapsulation efficiency for total polyphenols could be ascribed to very low encapsulation of neochlorogenic acid. In the literature, there are different results for this parameter, depending on wall materials, cross-linking solution, as well as the polyphenol profile of the encapsulation material and all studies are pointing out that optimization of encapsulation parameters was of main importance. The broad range of the encapsulation efficiencies of polyphenols of chokeberry extract in alginate beads was determined by Tzatsi and Goula [27] and their results varied from 8.94% to 93.87%. Encapsulation efficiency of total polyphenols and proanthocyanidins from pomegranate peel extract into alginate beads was from 32% to 46% depending on the initial concentration of polyphenols [21]. Stoica et al. [28] reported that encapsulation efficiency of rose hips polyphenols in alginate beads and alginate/chitosan beads ranged from 40% to 90%. When grape seed polyphenols were encapsulated in alginate and alginate/chitosan beads encapsulation efficiency was 78% and 92% [29]. Reported efficiency values by Pasukamonset et al. [30] were from 75 to 85% for polyphenols from extracts of *Clitoria ternatea* petal flower in the alginate beads. In the study by Li et al. [31], the authors tried to improve the encapsulation efficiency of tea polyphenols into alginate beads by inulin, Arabic gum or chitosan and observed that efficiency was 38.51%, 36.48%, 48.56% and 57.76%, respectively.

### 2.3. Evaluation of Antioxidant Activity

From the results of antioxidant activity (Table 2), it can be concluded that the addition of pectin caused its increase, regardless of the method used. The values of antioxidant activity determined by FRAP assay ranged from 2.72–3.68 µmol/100 g. Hydrogel beads containing only alginate as wall material had the lowest antioxidant activity level, while the addition of pectin caused its increase and ALG/PEC-30 had the highest value of antioxidant activity determined by this method. Prolonged complexation time also affected the antioxidant activity of the samples, i.e., it caused a decrease. The higher antioxidant activity could be achieved by a proper set of calcium to alginate ratios. If the membrane contains more alginate and less calcium it could ensure better protection of the antioxidants. This was also associated with gel strength since the high concentrations of calcium in an aquatic environment change the hydration process and the ability to cross-link and form gel decreases [32]. Moreover, studies indicated that a low concentration of alginate might result in the scarcity of carboxyl groups which are required to form the beads with calcium ions mass [30]. Antioxidant activity determined with CUPRAC assay revealed the same trend and it ranged from 176.56 µmol/100 g for ALG-90, to 230.49 µmol/100 g for ALG/PEC-30. From these results, it can be concluded that prolonged complexation did not affect positively the antioxidant activity of chokeberry hydrogel beads determined with CUPRAC method regardless of the wall material composition. For hydrogel beads, antioxidant activity determined with DPPH assay ranged from 22.56–28.24 µmol/100 g. Results obtained with this method showed that only the addition of pectin caused changes in antioxidant activity, while prolonged complexation did not cause any significant differences. Tzatsi and Goula [27] evaluated the antioxidant activity of chokeberry encapsulates prepared with different encapsulation techniques (spray-drying, co-crystallization and ionic gelation) with the DPPH method. The highest antioxidant activity (93.14%) was observed for beads prepared by ionic gelation, while the lowest was for spray-dried encapsulates (74.20%). It was reported that pectin interfered with the synergistic effect of polyphenols, modifying electron transfer in antioxidant activity determined by DPPH and FRAP methods [33]. As was the case with other methods, pectin addition positively affected the antioxidant activity of hydrogel beads determined with ABTS assay, and those values ranged from 25.37 µmol/100 g for hydrogel beads with only alginate as wall material to 35.41 µmol/100 g for those containing both polysaccharides. Prolonged complexation caused a decrease in antioxidant activity only in the case where the wall material was composed of alginate.

Due to the importance of the antioxidant defense, various methods for the determination of antioxidant activity were developed. Different methods are based on different principles and thus antioxidant activity should be tested based on several methods and one method cannot be compared to another. The information related to the hydrogen atom transfer or donating capacity of the electrons provides important information about the antioxidant potential of the samples with minimal environmental interference [34]. Another justification for applying different methods of analysis lies in the fact that methods can be easily influenced by various factors, as is the case with the DPPH method, where the presence and the concentration of hydrogen atoms and their activity corresponds to the number and position of hydroxyl groups in the aromatic ring of the compounds present. This could lead to misinterpretation of the results since the food is a very complex matrix [35].

Table 4 presents the calculated correlation coefficients for the dependence of antioxidant activity and the sum of concentrations of individual anthocyanins, phenolic acids and flavonols determined with HPLC method. The highest R^2^ values represent the highest correlation of polyphenols to antioxidant activities. Regardless of the method used to determine antioxidant activity, total anthocyanins were the most appropriate predictor of antioxidant activity (R^2^ was 0.9358, 0.8756, 0.8951 and 0.9532 for FRAP, CUPRAC, DPPH, ABTS, respectively), followed by correlation coefficient for total phenolic acids, which was satisfactory (R^2^ > 0.6554). It was observed that total flavonols showed a lack of correlation with antioxidant activity. Total anthocyanins as a good predictor of antioxidant activity were also reported in other studies [36,37].

### 2.4. Evaluation of Volatiles

Volatile compounds in chokeberry juice and hydrogel beads were divided into several groups; alcohols, acids, carbonyl compounds, esters, and terpenes. Their properties and flavor notes are presented in Table 5.

Amounts of volatiles in hydrogel beads (Table 6) depended on the composition of wall material and in some cases on the time of complexation. From the alcohol group, 7 volatiles were determined. Amounts of 3-hexen-1-ol and 1-hexanol were determined in higher amounts in ALG hydrogel beads. Phenetyl alcohol was determined only in ALG-30, thus prolonging complexation and addition of pectin did not cause encapsulation of this volatile. 1-Nonanol was found in significantly higher amounts in ALG/PEC hydrogel beads than in ALG hydrogel beads. Interestingly, this volatile was determined in ALG-30 beads while prolonged complexation prevented its encapsulation. The composition of hydrogel beads and time of complexation did not affect octanol and perillyl alcohol, i.e., efficiency of their encapsulation was identical. For 2-ethylhexanol, it was determined that after 30 min of complexation there was no difference between ALG and ALG/PEC hydrogel beads. With the prolonged complexation up to 90 min, encapsulation efficiency of this volatile increased and a higher amount was determined in ALG hydrogel beads. Three acids were determined. ALG beads had a higher amount of 2-ethylhexanoic acid than ALG/PEC ones. For nonanoic and decanoic acids it was determined that there was no difference between ALG hydrogel beads. However, for ALG/PEC hydrogel beads, it was determined that, with prolonged complexation, a decrease in encapsulation efficiency of both acids occurred. From carbonyl compounds, eight of them were detected in hydrogel beads. Octanal was the only aldehyde that was not detected in chokeberry juice and comparing both types of hydrogel beads it was determined in higher amounts in ALG/PEC hydrogel beads. The highest amount of benzaldehyde was in ALG-30 and after complexation for 90 min; this volatile was not encapsulated. In ALG/PEC hydrogel beads, it was determined in both hydrogels regardless of complexation time but in a lower amount when complexation was 90 min. For ALG/PEC hydrogel beads, the same trend was observed for octanal, while for ALG hydrogel beads the reverse trend was observed. Nonanal was evaluated in higher amounts in alginate/pectin hydrogel beads and with prolonged complexation, a higher amount of this volatile was encapsulated. The amount of decanal in ALG samples was equal, regardless of complexation time. ALG/PEC hydrogel beads had higher encapsulation efficiency for this volatile, which was more pronounced when complexation was only 30 min. Additionally, ALG/PEC hydrogel beads had higher encapsulation efficiency for geranylacetone, lilial and hexyl cinnamal. Myristaldehyde was determined in equal amounts in all hydrogel beads.

Five esters were determined in chokeberry juice, while in hydrogel beads two of them (ethyl hexanoate and hexyl acetate) were not detected. The higher encapsulation efficiency was evaluated for methyl dihydrojasmonate for ALG beads, while for phenethyl acetate there was no significant difference between hydrogels. Nine terpenes were detected in samples. Linalool, phellandral, β-damascenone and α-ionone were determined in equal amounts in all hydrogels, thus hydrogel composition and complexation time had no effect on these volatiles during the encapsulation process. The amount of D-limonene was the highest in ALG-30, while there was no difference between all the other hydrogels. Linalool oxide had the highest amount in ALG/PEC hydrogel beads when complexed for 90 min, while for ALG beads there was no difference between complexation times. γ-ionone and β-ionone were evaluated in higher amounts in ALG, while α-cedrol had the highest levels in the ALG/PEC hydrogel beads.

It was reported that the volatile profiles of fruits are affected by the addition of hydrocolloids by two possible mechanisms. One is due to the increased viscosity of the system which consequently lowers the diffusion rate of volatiles to the environment and the other is the binding of volatile compounds to the hydrocolloids [38]. Chemical groups as well as the assembly of chemical properties of volatile compounds are the most important factors determining their retention [39]. In the study of Misharina et al. [40] the binding of volatile compounds to alginate and other hydrocolloids (corn starch, carrageenan, xanthan, maltodextrin, carboxymethylcellulose, guar gum, citrus fiber, gum Arabic and locust bean gum) was investigated. It was observed that alginate bound mono- and sesquiterpenes, alcohols and ketones at a medium rate, which was similar to xanthan gum, citrus fiber, and maltodextrin. Another study revealed that, after the formation of alginate/gelatin hydrogel beads with black pepper essential oil, the D-limonene content was 12.10% and the decrease in all terpenes percentages was explained by their volatility and sensitivity to the external factors, which probably contributed to their reduction during the production of the beads. In this study, linalool was well preserved in hydrogel beads which is the opposite of the results obtained in the Heckert Bastos et al. [12] study. For the production of hydrogel beads with higher loading of volatile compounds, the application of concentric nozzles was proposed. The method is based on the flow of the polymer solution and volatile compounds simultaneously through internal and external nozzles and is added dropwise into the hardening bath [41]. The addition of pectin also had a certain effect on the encapsulation of volatiles. Pectin reacts with volatile molecules or forms hydrophobic micelles to capture them. These micelles are more hydrophobic and more nonpolar compounds may be captured in the hydrophobic parts of the pectin solution. In the study of Zhang and Barringer [42], linalool and 1-hexanol, as well as other strawberry volatiles were preserved by the addition of pectin. The same compounds originating from chokeberry juice were preserved in hydrogel beads prepared for the purpose of this research. Increasing the concentration of pectin is expected to preserve more volatile compounds due to the presence of more binding sites [43]. Except for hydrocolloid type and concentration, the texture of the hydrogels also has a considerable effect on the release of volatile compounds [44].

**Figure 1:**
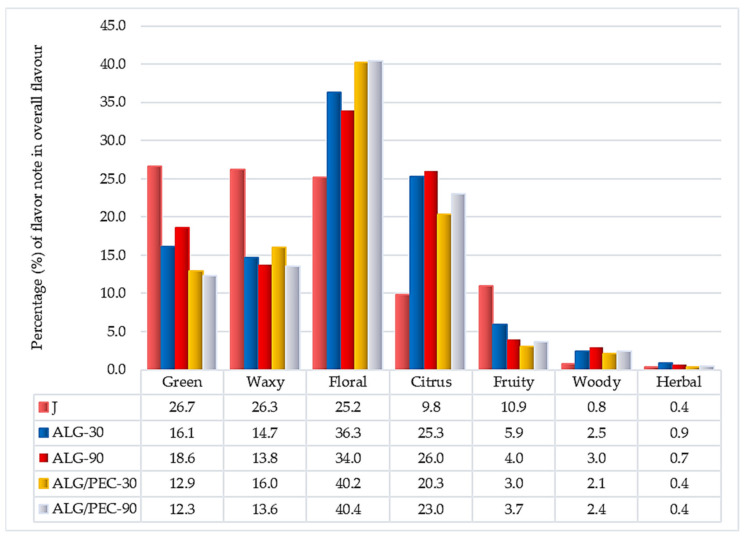
Percentage of flavor notes in the overall flavor of chokeberry juice and hydrogel beads (J: chokeberry juice, ALG, ALG/PEC: alginate, alginate/pectin as wall material(s), 30 and 90: different duration (minutes) of complexation. Alginate to pectin ratio was 2:1).

Each volatile compound has its characteristic flavor note, therefore based on this property, the contribution of each flavor note to the overall flavor of samples was determined (Figure 1). Green, floral and waxy flavor notes were the dominant ones in chokeberry juice, each contributed 26% to the overall flavor of the juice. Citrus and fruity flavor notes both contributed 10% to the overall flavor. Woody and herbal notes contributed under 1%. Prepared hydrogel beads had significantly different flavor profiles. In hydrogel beads, the most dominant flavor note was floral, which was more pronounced in ALG/PEC (40%) than in ALG (35%) beads. Citrus flavor notes contributed 26% to the overall flavor of ALG hydrogel beads and 22% to ALG/PEC hydrogel beads. Green flavor notes were higher in ALG beads (17%) than in ALG/PEC (12.5%). The same tendency was observed for fruity flavor notes (6.5% and 3.5%, respectively).

## 3. Conclusions

In the present study, microencapsulation of polyphenols and volatiles from chokeberry juice was applied, and the effects of the wall material composition and complexation time with the hardening solution on the polyphenol and volatile profile were investigated. Results revealed that alginate hydrogel beads combined with the pectin addition contained higher concentrations of polyphenols compared to those prepared with only alginate, except for some of the individual polyphenols determined by the HPLC method, which showed a slightly different trend. Encapsulation of volatiles also depended on the composition of hydrogel beads, and, in some cases, on the time of complexation. This investigation demonstrated that hydrogel beads can serve as a potential matrix for the microencapsulation of bioactive compounds and thus ensure preservation of their positive properties, such as antioxidant activity. However, the proper formulation of wall material is important because its interactions with polyphenols or volatiles have an impact on the retention of these valuable compounds. After preparation, hydrogel beads may be used as an ingredient of functional foods since components used for their production are nutritionally rich and safe for consumption.

## 4. Materials and Methods

### 4.1. Chemicals

Chokeberry fruits were bought at the local market. Alginic acid sodium salt (very low viscosity) was obtained from Alfa Aesar (Kandel, Germany), while low-methoxyl pectin (Genu pectin type LM-5-CS) was from CP Kelco (Lille Skensved, Denmark). Calcium chloride, ethanol, sodium acetate, potassium chloride and ammonium acetate were from Gram-mol (Zagreb, Croatia). Sodium carbonate was purchased from T.T.T. (Sveta Nedelja, Croatia). Potassium persulfate and Folin-Ciocalteu reagent were procured from Kemika (Zagreb, Croatia). Cupric chloride, neocuproine and 2,4,6-tri(2-pyridyl)-s-triazine (TPTZ) were bought from Acros Organic (Geel, Belgium). Methanol (HPLC grade) was from J.T. Baker (Deventer, The Netherlands) and orthophosphoric acid (HPLC grade, >85%) was from Fisher Scientific (Loughborough, UK). Hydrochloric acid (37%) and methanol were bought from Carlo Erba Reagents (Sabadell, Spain) and acetic acid (>99.5%) was from Alkaloid (Skopje, North Macedonia). Trolox, 2,2-diphenyl-1-picrylhydrazil, 2,2′-azino-bis(3-ethylbenzothiazoline-6-sulfonic acid) diammonium salt, 4-dimethyl-amino-cinnamaldehyd and most of the standards used for HPLC analysis (chlorogenic acid, quercetin and rutin) were from Sigma-Aldrich (St. Louis, MO, USA), while the standards of cyanidin-3-galactoside, neochlorogenic acid and hyperoside were products of Extrasynthese (Genay, France).

### 4.2. Preparation of Hydrogel Beads

Chokeberry juice was prepared by pressing the fruits and filtering the obtained mass. The juice (pH value 3.78) was then used for the preparation of encapsulation mixture in which 2% of alginate was dissolved by stirring using a stick blender (pH value 3.90). Another set of samples was prepared by adding 1% of pectin to the alginate-chokeberry juice mixture (pH value 3.88). After all components in the encapsulation mixture have been dissolved, they were complexed for 30 min before performing the microencapsulation process. As a hardening solution, 7% CaCl_2_ in chokeberry juice (pH value 2.88) was used. Hydrogel beads were formed using Encapsulator B-390 (BÜCHI Labortechnik AG, Flawil, Switzerland) under fixed conditions: 1000 µm vibrating nozzle, pressure 200 mbar, frequency 200 Hz and electrode 1000 V. After the formation of beads, they were left in a hardening solution for 30 or 90 min to investigate whether the time of complexation of hydrogel beads with CaCl_2_ had an impact on the preservation of bioactives from chokeberry juice. Produced hydrogel beads were then filtered and used for further analyses.

### 4.3. Extraction of Polyphenols from Hydrogel Beads

The method of Kopjar et al. [45] for extraction of polyphenols from hydrogel beads was used with slight modifications. Approximately 1.5 g of hydrogel beads were weighed, and 10 mL of acidified methanol (the ratio of methanol to hydrochloric acid was 99:1) was added. Then the samples were placed in an ultrasonication bath (Bandelin sonorex digitec, Berlin, Germany) for 10 min, followed by insertion into a vortex (MSV-3500, Biosan, Riga, Latvia) for 2 h at a rotational speed of 800 rpm. The mixture was filtered and the extracts were used for spectrophotometric and HPLC evaluations.

### 4.4. Spectrophotometric Determination of Total Polyphenols, Monomeric Anthocyanins and Antioxidant Activity in Extracts

#### 4.4.1. Total Polyphenols and Monomeric Anthocyanins

Total polyphenols were determined using the method by Singleton and Rossi [46] with slight modifications. Firstly, 0.2 mL of extract was added to the glass tube followed by the addition of 1.8 mL of deionized water, 10 mL of Folin-Ciocalteu reagent (1:10) and 8 mL of sodium carbonate (7.5%). Prepared mixtures were kept in the dark. After 120 min, the absorbance of samples was measured at 765 nm using a spectrophotometer (Cary 60, UV-VIS, Agilent Technologies, Santa Clara, CA, USA). Each sample was analyzed in triplicate. After interpolation on a gallic acid calibration curve, results were expressed as mg of gallic acid equivalents per g of hydrogel beads (mg GAE/g).

Determination of monomeric anthocyanins content was carried out using the pH differential method [47]. Briefly, 2.8 mL of 0.025 M KCl at pH 1 or 2.8 mL of 0.4 M sodium acetate at pH 4.5 were added to 0.2 mL of extract and the mixtures were kept in the dark for 15 min. Each sample was analyzed in triplicate. Absorbance was calculated using the equation:

(1) $$A={\left(A_{515}-A_{700}\right)}_{\mathrm{pH}\;1\;}-{\left(A_{515}-A_{700}\right)}_{\mathrm{pH}\;4.5}$$

where A_515_ represents absorbance read at 515, while A_700_ is the absorbance read at 700 nm. The concentration of monomeric anthocyanins was calculated as follows:

(2) $$\mathrm{Monomeric}\;\mathrm{anthocyanins}=\left({A\;\times \;M}_{W}\;\times \;\mathrm{DF}\;\times \;1000\right)/(\varepsilon \;\times \;l)$$

where M_W_ represents the molecular weight of cyanidin-3-glucoside (449.2), DF is the dilution factor, ε is the molar absorptivity (26,900) and l is the cuvette length (1 cm). Since the concentration of monomeric anthocyanins was calculated using data for cyanidin-3-glucoside, their contents were expressed as mg of cyanidin-3-glucoside per gram of beads (mg cyanidin-3-glucoside/g).

#### 4.4.2. Determination of Antioxidant Activity (FRAP, CUPRAC, DPPH and ABTS Assays)

Firstly, to determine ferric reducing ability extract (0.2 mL) was added to FRAP reagent (3 mL). The mixture was left in the dark for 30 min and absorbance was read at 593 nm. This method was from Benzie and Strain [48]. Secondly, copper (II) reducing antioxidant activity or CUPRAC assay was conducted using the method previously described by Apak et al. [49]. To conduct the analysis, 1 mL of copper chloride, necuproine and ammonium acetate buffer (at pH 7) were mixed in the given order and then 0.2 mL of extract and 0.9 mL of distilled water were added to the mixture. Absorbance was read at 450 nm after 30 min of incubation. Thirdly, the DPPH assay was carried out according to Brand-Williams et al. [50]. DPPH solution (0.5 mM) was prepared and 3 mL was added to 0.2 mL of extract. The mixture was left in the dark for 15 min and after that, absorbance was measured at 517 nm. Finally, the method of Arnao et al. [51] was adapted for the determination of antioxidant activity using ABTS reagent. In short, 3.2 mL of reagent was added to 0.2 mL of the sample and absorbance was measured at 734 nm after 95 min.

For the determination of antioxidant activity, each sample was analyzed in triplicate and the calibration curve was created for Trolox. Results were expressed as µmol of Trolox equivalents per 100 g of hydrogel beads (µmol TE/100 g).

### 4.5. High-Performance Liquid Chromatography (HPLC) for Evaluation of Individual Polyphenols

Agilent HPLC system 1260 Infinity II (Agilent technology, Santa Clara, CA, USA) was used to evaluate the individual polyphenols in extracts and chokeberry juice. The system consisted of a quaternary pump, a vial sampler, Poroshell 120 EC C-18 column (4.6 × 100 mm, 2.7 µm) and a diode array detector (DAD). Before injecting into the system, extracts were filtered through PTFE filters with 0.20 µm pores. The method used was previously described by Buljeta et al. [52]. The injection volume was 10 µL and the flow rate was 1.0 mL/min. UV-Vis spectra were recorded in the wavelength range from 190 to 600 nm. Orthophosphoric acid (0.1% water solution) was used as a mobile phase A and methanol (100%) was used as the mobile phase B. The gradient used for separation was set as follows: 0 min 5% B, 3 min 30% B, 15 min 35% B, 22 min 37% B, 30 min 41% B, 32 min 45% B, 40 min 49% B, 45 min 80% B, 48 min 80% B, 50 min 5% B, 53 min 5% B. Identification of polyphenols was carried out by comparing the retention times and the UV-Vis spectra of peaks in the extracts. The calibration curve for cyanidin-3-galactoside was obtained at a concentration range from 5–300 mg/L, for chlorogenic acid from 25–500 mg/L, for neochlorogenic acid from 1–300 mg/L, for quercetin and hyperoside from 5–150 mg/L and for rutin from 0.25–500 mg/L. The linearity of the curves was confirmed by R^2^ = 0.9969 for cyanidin-3-galactoside, R^2^ = 0.999 for chlorogenic acid, R^2^ = 0.9986 for neochlorogenic acid, R^2^ = 0.9998 for quercetin, R^2^ = 0.9993 for hyperoside and R^2^ = 0.9993 for rutin. Cyanidin-3-arabinoside and cyanidin-3-xyloside were detected tentatively with the help of literature data [53,54]. Their concentrations were calculated using a calibration curve created for cyanidin-3-galactoside. Two injections were performed from each prepared solution. The profile of polyphenols in the samples was expressed as mg of polyphenols per kg of hydrogel beads or chokeberry juice (mg/kg).

### 4.6. Volatile Compounds Analysis

Extraction of volatile compounds from hydrogel beads was conducted using solid-phase microextraction (SPME) and fiber coated with divinylbenzene/carboxen/polydimethylsiloxane (DVB/CAR/PDMS) sorbent (50/30 µm, StableFlex™, Supelco, Bellefonte, PA, USA). Firstly, 0.3 g of sample, 4.7 g of water and 1 g of NaCl and were weighed in glass vials, which were then conditioned in a temperature-controlled heating module under the following conditions: 40 °C, 45 min and 350 rpm. After extraction, fiber was removed from samples and volatiles were thermally desorbed in the injector port of the GC 7890B gas chromatograph (Agilent Technologies, Santa Clara, CA, USA) which was used to perform the analysis. It was equipped with a 5977A mass spectrometer (Agilent Technologies, Santa Clara, CA, USA). Volatiles were desorbed into the GC injector port at 250 °C for 7 min in a splitless mode. The gas chromatograph was fitted with an HP5 capillary column (60 m × 0.25 mm × 0.25 µm). Helium was used as the carrier gas, and its flow rate was set at 1 mL/min and 40 °C. The initial temperature of the oven was 40 °C and it was held for 2 min and then the temperature was set to rise at 6 °C/min up to 230 °C. For the identification of volatiles, mass selective detector was used and operated in the range of *m*/*z* between 45 and 450. Ion source temperature was 230 °C and the temperature of the quadrupole was maintained at 150 °C. Confirmation of the compounds was performed by matching their mass spectra with the NIST (National Institute of Standards and Technology mass spectral database, Gaithersburg, MD, USA) and through retention time (RT) and retention index (RI) (Table 5). For the quantification of compounds, myrtenol was used as an internal standard. Each sample was analyzed twice and the results were expressed as µg/100 g.

### 4.7. Statistical Analysis

All results were expressed as the mean values ± standard deviation. Statistical analysis was performed using software STATISTICA 13.1 (StatSoft Inc., Tulsa, OK, USA). Analysis of the variance (ANOVA) and Fisher’s least significant difference (LSD) with the significance defined at *p* < 0.05 were used for data analysis.

## Figures and Tables

**Table 1:** Evaluation of chokeberry juice polyphenols and antioxidant activity.

Antioxidant Activity (µmol/100 g)	
FRAP	2.60 ± 0.00
CUPRAC	166.63 ± 0.14
DPPH	21.36 ± 0.21
ABTS	36.07 ± 0.15
Concentrations of Individual Polyphenols (mg/kg)	
Anthocyanins	
Cyanidin-3-galactoside	41.68 ± 0.58
Cyanidin-3-arabinoside	10.31 ± 0.22
Cyanidin-3-xyloside	1.11 ± 0.00
Phenolic acids	
Chlorogenic acid	172.07 ± 0.47
Neochlorogenic acid	1409.75 ± 34.44
Flavonols	
Quercetin	3.21 ± 0.00
Hyperoside	9.61 ± 0.01
Rutin	25.54 ± 0.03

DPPH (2,2-diphenyl-1-picrylhydrazyl) - free radical scavenging activity; ABTS (2,20 -azino-bis(3-ethylbenzothiazoline-6-sulfonic acid) - radical scavenging activity; FRAP–ferric reducing antioxidant power; CUPRAC–cupric reducing antioxidant capacity.

**Table 2:** Total polyphenols, anthocyanins and antioxidant activity of hydrogel beads.

Sample	Total Polyphenols (g/kg)	Monomeric Anthocyanins (g/kg)	FRAP (µmol/100 g)	CUPRAC (µmol/100 g)	DPPH (µmol/100 g)	ABTS (µmol/100 g)
ALG-30	4.51 ± 0.29 ^a^	1.42 ± 0.00 ^c^	3.06 ± 0.00 ^b^	183.81 ± 0.42 ^b^	22.95 ± 0.08 ^a^	27.63 ± 0.31 ^b^
ALG-90	4.36 ± 0.14 ^a^	1.29 ± 0.01 ^a^	2.72 ± 0.00 ^a^	176.56 ± 0.40 ^a^	22.14 ± 0.21 ^a^	25.37 ± 0.64 ^a^
ALG/PEC-30	5.63 ± 0.17 ^b^	1.34 ± 0.01 ^b^	3.68 ± 0.00 ^d^	230.49 ± 0.35 ^d^	28.59 ± 0.79 ^b^	35.24 ± 0.08 ^c^
ALG/PEC-90	5.69 ± 0.18 ^b^	1.47 ± 0.01 ^d^	3.49 ± 0.00 ^c^	220.05 ± 0.47 ^c^	27.89 ± 0.46 ^b^	35.58 ± 0.26 ^c^

ALG, ALG/PEC: alginate, alginate/pectin as wall material(s), 30 and 90: different duration (minutes) of complexation. Alginate to pectin ratio was 2:1. Within the column, means followed by different superscript letters are significantly different at *p* ≤ 0.05 (ANOVA, Fisher’s LSD).

**Table 3:** Concentrations of individual polyphenols in hydrogel beads determined using the HPLC method.

Sample	Anthocyanins (mg/kg)		Phenolic Acids (mg/kg)		Flavonols (mg/kg)		
	C3G	C3A	CHA	NCHA	Q	H	R
ALG-30	91.87 ± 0.09 ^b^	23.10 ± 0.00 ^a,b^	303.09 ± 1.09 ^a^	181.60 ± 2.69 ^b^	24.82 ± 0.08 ^a^	24.91 ± 0.08 ^a,c^	36.70 ± 0.04 ^a^
ALG-90	87.86 ± 0.48 ^a^	22.06 ± 0.08 ^a^	309.40 ± 2.95 ^a^	167.75 ± 5.31 ^b^	25.00 ± 0.06 ^a^	24.24 ± 0.08 ^a^	34.68 ± 0.08 ^a^
ALG/PEC-30	95.78 ± 2.46 ^b^	24.73 ± 0.57 ^b,c^	317.54 ± 1.05 ^b^	144.38 ± 6.16 ^a^	25.86 ± 0.02 ^b^	24.83 ± 0.23 ^a,b^	34.76 ± 1.06 ^a^
ALG/PEC-90	96.50 ± 2.02 ^b^	24.91 ± 0.62 ^c^	322.34 ± 0.36 ^b^	140.17 ± 4.81 ^a^	26.08 ± 0.21 ^b^	25.01 ± 0.26 ^b,c^	35.78 ± 0.84 ^a^

ALG, ALG/PEC: alginate, alginate/pectin as wall material(s), 30 and 90: different times (minutes) of complexation. Alginate to pectin ratio was 2:1. C3G—cyanidin-3-galactoside; C3A—cyanidin-3-arabinoside; CHA—chlorogenic acid; NCHA—neochlorogenic acid; Q—quercetin; H—hyperoside; R—rutin. Within the column, means followed by different superscript letters are significantly different at *p* ≤ 0.05 (ANOVA, Fisher’s LSD).

**Table 4:** Calculated correlation coefficients (R^2^) which describe the dependence of antioxidant activities and the sum of concentrations of anthocyanins, phenolic acids and flavonols determined with HPLC method.

R^2^	FRAP	CUPRAC	DPPH	ABTS
Total anthocyanins	0.9358	0.8756	0.8951	0.9532
Total phenolic acids	0.6554	0.8387	0.8605	0.8048
Total flavonols	0.3326	0.1960	0.2219	0.3341

**Table 5:** Physicochemical properties, flavor notes and amount of volatiles (J) in chokeberry juice.

Volatiles	RT	RI	MW	logP (o/w)	VP (mm/Hg)	J (µg/100 g)	Flavor Note
3-hexen-1-ol	7.5448	849	100.16	1.697	1.039	66.34 ± 5.31	Green
1-hexanol	8.3815	868	102.18	2.03	0.947	933.50 ± 11.96	Green
Benzaldehyde	14.7581	955	106.12	1.480	1.270	58.10 ± 0.28	Fruity
Octanal	17.9018	997	128.21	2.951	2.068	-	Green
Ethyl hexanoate	18.0561	998	144.21	2.823	1.665	34.12 ± 0.33	Fruity
Hexyl acetate	18.7547	1009	144.21	2.870	1.391	319.63 ± 0.10	Fruity
D-limonene	19.1852	1018	136.24	4.570	0.198	103.09 ± 5.69	Citrus
2-ethylhexanol	19.6808	1030	130.23	2.820	0.207	188.52 ± 1.40	Citrus
Linalool oxide	21.9958	1068	170.25	1.375	0.002	12.42 ± 0.32	Floral
Octanol	22.2558	1071	130.23	3.000	0.079	114.97 ± 2.13	Green
Linalool	23.7423	1096	154.25	2.970	0.016	41.01 ± 0.57	Citrus
Nonanal	23.9941	1095	142.24	3.461	0.532	130.10 ± 0.00	Citrus
Phenethyl alcohol	24.3597	1103	122.17	1.360	0.087	365.46 ± 2.15	Floral
2-ethylhexanoic acid	26.1874	1128	144.21	2.640	0.030	18.44 ± 1.82	Herbal
1-nonanol	27.8932	1168	144.26	3.770	0.041	260.29 ± 4.01	Waxy
Decanal	29.396	1200	156.27	3.970	0.207	209.75 ± 11.62	Floral
Phenethyl acetate	31.8005	1250	164.20	2.300	0.056	182.06 ± 3.10	Floral
Phellandral	32.4665	1264	152.24	3.168	0.098	72.90 ± 0.95	Floral
Nonanoic acid	33.1976	1277	158.24	3.42 0	0.009	310.23 ± 5.87	Waxy
Perillyl alcohol	33.7338	1286	152.24	2.100	0.006	154.10 ± 3.09	Green
Decanoic acid	37.4785	1376	172.27	4.090	15.00	679.29 ± 6.97	Waxy
β-damascenone	37.6004	1377	190.29	4.042	0.020	55.55 ± 0.13	Floral
Ethyl decanoate	37.9984	1391	200.32	4.861	0.034	38.67 ± 1.43	Fruity
α-ionone	38.8269	1417	192.30	3.995	0.014	18.64 ± 2.15	Fruity
Geranylacetone	39.5093	1448	194.32	3.834	0.016	181.48 ± 0.29	Floral
γ-ionone	40.0779	1470	192.30	3.505	0.008	21.51 ± 2.13	Fruity
β-ionone	40.2323	1477	192.30	3.995	0.017	29.54 ± 0.14	Fruity
Lilial	40.0528	1517	204.31	4.216	0.005	42.35 ± 0.64	Floral
α-cedrol	42.3443	1592	222.37	4.330	0.001	21.51 ± 3.48	Woody
Myristaldehyde	42.4661	1601	212.38	6.008	0.006	14.70 ± 0.72	Woody
Methyl dihydrojasmonate	43.1078	1644	226.32	2.653	0.001	43.97 ± 2.20	Floral
Hexyl cinnamal	44.3507	1738	216.32	4.866	0.001	31.85 ± 0.46	Floral

>RT—retention time, RI—retention index, MW—molecular weight, logP—hydrophobicity, VP—vapor pressure at 25 °C, J—chokeberry juice.

**Table 6:** Amount (µg/100 g) of volatile compounds in chokeberry juice and hydrogel beads containing chokeberry juice.

Volatiles	ALG-30	ALG-90	ALG/PEC-30	ALG/PEC-90
Alcohols	164.1	174.9	175.4	171.6
3-hexen-1-ol	9.85 ± 0.14 ^b^	10.56 ± 0.03 ^b^	6.68 ± 0.00 ^a^	6.66 ± 0.43 ^a^
1-hexanol	24.85 ± 0.04 ^d^	23.11 ± 0.36 ^c^	9.33 ± 1.03 ^b^	5.33 ± 0.96 ^a^
2-ethylhexanol	67.58 ± 0.04 ^a^	83.17 ± 1.17 ^c^	68.73 ± 1.32 ^a^	71.86 ± 0.11 ^b^
Octanol	13.40 ± 0.54 ^a^	15.63 ± 3.13 ^b^	15.44 ± 0.07 ^b^	15.00 ± 1.95 ^b^
Phenethyl alcohol	3.95 ± 0.00 ^a^	-	-	-
1-nonanol	7.58 ± 0.41 ^a^	-	32.37 ± 0.83 ^b^	32.83 ± 2.10 ^b^
Perillyl alcohol	36.86 ± 0.32 ^a^	42.39 ± 2.39 ^c^	42.87 ± 1.27 ^c^	39.95 ± 0.42 ^b^
Acids	89.9	90.1	106.6	76.6
2-ethylhexanoic acid	5.34 ± 0.39 ^b^	4.47 ± 0.74 ^b^	3.35 ± 0.33 ^a^	3.50 ± 0.11 ^a^
Nonanoic acid	16.28 ± 0.57 ^a^	19.95 ± 1.14 ^a^	32.78 ± 0.53 ^b^	16.40 ± 2.53 ^a^
Decanoic acid	68.26 ± 0.91 ^b^	65.68 ± 1.55 ^b^	70.49 ± 1.50 ^b^	56.70 ± 0.39 ^a^
Carbonyl compounds	238.9	229.9	394.5	362.7
Benzaldehyde	6.93 ± 0.31 ^c^	-	2.71 ± 0.18 ^b^	1.91 ± 0.16 ^a^
Octanal	15.80 ± 1.33 ^a^	23.39 ± 0.70 ^b^	34.79 ± 2.33 ^c^	29.10 ± 1.52 ^c^
Nonanal	37.82 ± 2.26 ^a^	43.62 ± 1.22 ^b^	59.54 ± 0.85 ^c^	63.64 ± 1.91 ^d^
Decanal	81.84 ± 2.12 ^a^	85.95 ± 1.63 ^a^	161.50 ± 0.03 ^c^	126.98 ± 0.02 ^b^
Geranylacetone	58.46 ± 0.91 ^b^	39.04 ± 0.58 ^a^	88.77 ± 1.69 ^c^	92.70 ± 2.11 ^c^
Lilial	18.42 ± 0.38 ^b^	15.39 ± 0.37 ^a^	21.01 ± 0.63 ^c^	22.06 ± 0.50 ^c^
Myristaldehyde	9.48 ± 0.89 ^a^	10.62 ± 1.21 ^a^	10.37 ± 1.14 ^a^	10.26 ± 0.51 ^a^
Hexyl cinnamal	10.14 ± 0.15 ^a^	11.88 ± 0.34 ^b^	15.82 ± 0.35 ^c^	16.08 ± 0.42 ^c^
Esters	14.5	28.2	20.5	24.3
Ethyl hexanoate	-	-	-	-
Hexyl acetate	-	-	-	-
Phenethyl acetate	4.96 ± 0.61 ^a^	5.42 ± 0.11 ^a^	4.81 ± 0.88 ^a^	4.78 ± 0.32 ^a^
Ethyl decanoate	5.70 ± 0.86 ^b^	5.58 ± 0.14 ^b^	3.99 ± 0.27 ^a^	7.66 ± 0.47 ^c^
Methyl dihydrojasmonate	17.82 ± 0.79 ^b^	17.17 ± 1.29 ^b^	11.66 ± 0.67 ^a^	11.86 ± 0.77 ^a^
Terpenes	116.4	95.1	96.7	102.8
D-limonene	39.24 ± 2.91 ^b^	22.56 ± 2.49 ^a^	24.80 ± 0.36 ^a^	26.23 ± 1.51 ^a^
Linalool oxide	4.60 ± 0.32 ^b^	4.56 ± 0.07 ^b^	3.58 ± 0.12 ^a^	6.44 ± 0.80 ^c^
Linalool	10.92 ± 0.31 ^b^	9.80 ± 0.41 ^b^	8.29 ± 0.46 ^a^	8.26 ± 0.03 ^a^
Phellandral	25.50 ± 1.63 ^a^	25.78 ± 0.76 ^a^	28.24 ± 1.61 ^a^	28.79 ± 0.88 ^a^
β-damascenone	5.85 ± 0.02 ^a^	5.42 ± 0.64 ^a^	5.02 ± 0.33 ^a^	5.12 ± 0.03 ^a^
α-ionone	4.88 ± 0.70 ^b^	3.99 ± 0.45 ^a^	4.14 ± 0.30 ^a^	3.82 ± 0.27 ^a^
γ-ionone	9.21 ± 0.06 ^b^	6.38 ± 1.11 ^a^	8.56 ± 0.67 ^b^	8.74 ± 0.74 ^b^
β-ionone	10.30 ± 0.96 ^b^	8.71 ± 0.24 ^b^	6.41 ± 0.05 ^a^	6.75 ± 0.56 ^a^
α-cedrol	5.93 ± 0.10 ^a^	7.88 ± 2.17 ^b^	7.67 ± 0.24 ^b^	8.60 ± 0.49 ^b^

ALG, ALG/PEC: alginate, alginate/pectin as wall material(s), 30 and 90: different duration (minutes) of complexation. Alginate to pectin ratio was 2:1. Within the row, means followed by different superscript letters are significantly different at *p* ≤ 0.05 (ANOVA, Fisher’s LSD).

## Data Availability

Data is contained within the article.
